# The largest European forest carbon stocks are in the Dinaric Alps old-growth forests: comparison of direct measurements and standardised approaches

**DOI:** 10.1186/s13021-024-00262-4

**Published:** 2024-05-13

**Authors:** Alessia Bono, Giorgio Alberti, Roberta Berretti, Milic Curovic, Vojislav Dukic, Renzo Motta

**Affiliations:** 1 Department of Agricultural, Forest and Food Sciences (DISAFA), University of Turin, Largo Paolo Braccini 2 - IT, Grugliasco, TO 10095 Italy; 2 Department of Agricultural, Food, Animal and Environmental Sciences, University of Udine, Via delle Scienze 206 - IT, Udine, UD 33100 Italy; 3 Biotechnical Faculty, University of Montenegro, Mihaila Lalica 1, Podgorica, Montenegro; 4 University of Banja, Luka, blv. Stepa Stepanović, 75, Banja Luka, 78000 Republic of Srpska

**Keywords:** Abies alba, Biomass, Carbon pool, Carbon stock, Deadwood, Fagus sylvatica, Litter, Soil

## Abstract

**Background:**

Carbon (C) sink and stock are among the most important ecosystem services provided by forests in climate change mitigation policies. In this context, old-growth forests constitute an essential reference point for the development of close-to-nature silviculture, including C management techniques. Despite their small extent in Europe, temperate old-growth forests are assumed to be among the most prominent in terms of biomass and C stored. However, monitoring and reporting of C stocks is still poorly understood. To better understand the C stock amount and distribution in temperate old-growth forests, we estimated the C stock of two old-growth stands in the Dinaric Alps applying different assessment methods, including direct and indirect approaches (e.g., field measurements and allometric equations vs. IPCC standard methods). This paper presents the quantification and the distribution of C across the five main forest C pools (i.e., aboveground, belowground, deadwood, litter and soil) in the study areas and the differences between the applied methods.

**Results:**

We report a very prominent C stock in both study areas (507 Mg C ha^− 1^), concentrated in a few large trees (36% of C in 5% of trees). Moreover, we found significant differences in C stock estimation between direct and indirect methods. Indeed, the latter tended to underestimate or overestimate depending on the pool considered.

**Conclusions:**

Comparison of our results with previous studies and data collected in European forests highlights the prominence of temperate forests, among which the Dinaric Alps old-growth forests are the largest. These findings provide an important benchmark for the development of future approaches to the management of the European temperate forests. However, further and deeper research on C stock and fluxes in old-growth stands is of prime importance to understand the potential and limits of the climate mitigation role of forests.

## Background

Old-growth forests, according to the European Commission [1] and the Convention on Biological Diversity [2], are defined as “*stands in primary or secondary forests that have developed the structures and species normally associated with old primary forest of that type*” in which “*visible signs of anthropic activities are not present, and the ecological processes are not significantly disturbed*” [3].

In Europe, old-growth forests are estimated to cover 3.5 million ha (2.0% of the European forest area), with an uneven distribution among EU countries and forest types: about 90% of them are located in Sweden, Bulgaria, Finland, and Romania and more than 70% of them are located in the boreal forests [4, 5].

Even if most European old-growth forests have been protected, in some European regions, they are still declining at alarming rates, and their mapping and protection have been indicated among the priorities of the EU Biodiversity Strategy and Forest Strategy for 2030 [6]. The protection of old-growth forests is very important because they play a strategic role hosting a very peculiar biodiversity [7–9] and regulating water and nutrient cycling. Moreover, they are an essential reference for the application of a sustainable and closer to nature silviculture in managed forests and can guide the restoration of degraded forest ecosystems and store large amounts of carbon in absence of large-scale disturbances [10–16]. Due to the current climate change and emission reduction policies, carbon (C) sink and stock are noteworthy services provided by forests thanks to their capacity to absorb atmospheric carbon dioxide (CO_2_) and store C in alive and dead plant tissues and into the soil [17]. Consequently, C sequestration has become a recognized forest management objective [18, 19]. Anyway, only few studies about C stock in European old-growth forests are available, especially if we consider the temperate biomes [20–23].

Old-growth temperate forests of the northern hemisphere are rare (less than 1% of the forest cover) as temperate forests are generally more altered than other forested biomes because of their moderate climate and the long-term land use and competition with agriculture [24, 25]. Nevertheless, temperate old-growth forests are assumed to be among the most prominent in terms of C stored in the living and dead biomass [17, 26, 27]. Despite this, monitoring and reporting temperate old-growth forest C stocks has been based on default values of forest aboveground biomass (AGB) from the Intergovernmental Panel on Climate Change (IPCC) guidelines for National Greenhouse Gas (GHG) Inventories as well as on satellite data [28]. The validation of such estimates using site-specific allometric functions and direct field measurements are pretty limited [29, 30].

We have analysed two of the best preserved mixed montane old-growth forests in the Dinaric Alps of Bosnia-Herzegovina and Montenegro [31, 32] that are thought to be among the largest living biomass C stock in Europe [33, 34]. The final purpose of this paper was to compare different forest C stock indirect assessment methods with direct field measurements. Another purpose was to provide a C benchmark that could provide a reference for managed forests with the same species composition thus supporting a broad range of ecosystem functions and services. More specifically, we have assessed the C stock separately per ecosystem pool using direct field measurements and allometric relationships and, we have compared these results with estimates derived by applying the standard IPCC methodology [35] and the same IPCC method modified using different global allometric biomass equations [30].

## Methods

### Study sites

**Fig. 1:**
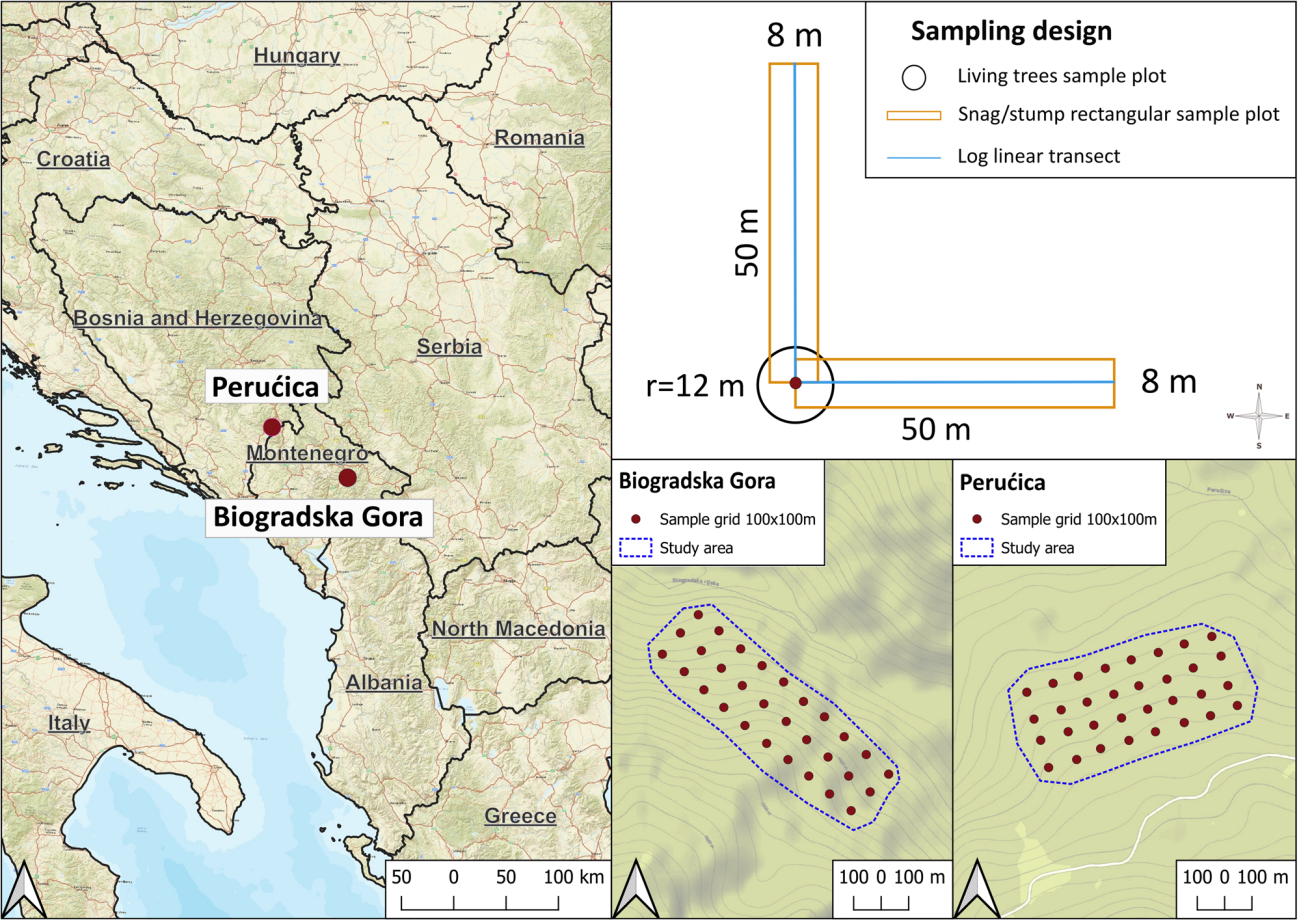
Localization of the two study areas (left) and the sampling design (right)

The study was conducted in two silver fir (*Abies alba* Mill.), beech (*Fagus sylvatica* L.) and spruce (*Picea abies* Karst.) mixed old-growth forests in the Dinaric Alps. The first site is located in Biogradska Gora (BGO) National Park in Montenegro, and the second one is located in Perućica (PER) national reserve within the Sutjeska National Park in Bosnia-Herzegovina [36]. At each site, a core study area of 33.1 and 35.2 ha, respectively in BGO and PER, was identified after intensive recognition (Fig. 1). This area in BGO has an average elevation of 1,453 m asl, and a south aspect. The mean annual precipitation at Biogradsko lake (1093 m a.s.l.) is 1,962 mm, with a mean annual temperature of 5 °C. The study area in PER has an average elevation of 1,363 m asl, and a south-west aspect. Climate is a mix of Mediterranean and Continental, with mean annual precipitation of 1,400 mm and mean annual temperature of 11.3 °C [37]. Both study sites are located in the core areas of old-growth sector of the national parks and are within the Global Ecological Zone (GEZ) of temperate mountain systems [38].

### Carbon estimation according to the data collected in the field

The forest carbon pools were assessed according to IPCC (2003) [35]. Five compartments were considered: living aboveground biomass (hereafter aboveground biomass), living belowground biomass (hereafter, belowground biomass), deadwood, litter, and soil.

### Aboveground biomass

The dendro-auxometric data were collected in 30 and 32 circular sample plots (*r* = 12 m) located according to a regular grid of 100 × 100 m in BGO and PER (Fig. 1), respectively. In each plot, species, diameter at breast height (DBH; to the nearest 0.01 m) and total height (to the nearest 0.1 m) were registered for each living tree (DBH > 7.5 cm). Site species-specific volume tables were used to estimate aboveground volume [39, 40]. The estimated volume was converted into biomass applying a basic wood density (D, Mg dry matter/ m^3^ fresh volume) of 0.40 for silver fir and spruce, 0.58 for beech and 0.52 for maples [35, 41]. The biomass was finally converted into C stock according to IPCC (2006) [42] using an average C content of 48% and 51% for broadleaves and conifers, respectively.

Leaf area index (LAI) was determined optically using a LAI2000 Plant Canopy Analyzer (Li-Cor Biosciences, Lincoln, NE) along a 100 m transect at each experimental site (one point every 2 m). Four shoots per species were sampled after LAI measurements in the middle third (by height) of trees’ crown. Leaves were separated from the shoot, collected, and stored in plastic bags. Once in the lab, the leaves were dried at 70 °C and then weighted. A fresh sub-sample of leaves was scanned, and total leaf area calculated using the ImageJ software and then dried in order to compute the specific leaf mass (g cm^− 2^) and convert the LAI (m^2^ m^− 2^) into biomass (Mg ha^− 1^), also taking into account the basal area fraction of each species. The sub-sampled leaves were finally reduced to sawdust for the determination of the C content using an elemental analyser (© Elementar Vario Microcube).

### Belowground biomass

Root C stock was estimated following two methods. The first allowed to calculate belowground biomass C starting by aboveground C stock using a root to shoot ratio of 0.20 and 0.24 for conifers and broadleaves, respectively [42, 43]. The second was based on an allometric equation proposed by Forrester et al. [30] which led to root biomass starting from trees DBH.

### Deadwood

Deadwood was classified in three main components: logs (lying dead trees and woody debris), snags (standing stems and dead trees) and stumps. Logs data were collected according to the line intercept method [44]: the diameter of each laying element (> 10 cm) intersecting a linear transect of 50 m was recorded. In each plot, two transects (northward and eastward) were considered. Snags and stumps data were gathered in a stripe transect (50 × 8 m) overlapped to log transect and centred in the centre of each plot (Fig. 1). Snag DBH (> 7.5 cm) and height and stump (dead stem < 130 cm height) diameter at the top (> 5 cm) and at the bottom as well as height were measured. Each deadwood element, was also classified by decay stage into a five-class system [45]. As for living trees, deadwood volume was converted to C mass through basic density (i.e., ratio between dry weight and fresh volume) and carbon content (%). Because these values are decay-dependent [46], for each decay class three sample were collected to determine the basic density. It was not possible to distinguish among species, especially at the most decomposed stages, anyway the range of species was very limited (silver fir, Norway spruce and beech). Once in the lab, fresh volume of the collected samples was measured by immersion in water before drying them at 102 °C for 48 h to measure dry weight. Samples were then reduced to sawdust and sub-samples were used for C content determination using a CHNS Elemental Analyser (Vario Microcube, © Elementar).

### Litter

Leaves and needles, seeds, fruits, and fine woody material (max diameter 1 cm) were collected within four randomly selected plots in a 1 × 1 m area. Material was stored at 4 °C and total fresh weight was recorded after arrival in the lab. Fresh weight of four sub-samples was also recorded. These samples were then dried at 70 °C to determine the dry weight. Total fresh litter weight was converted into dry biomass using the ratio between dry and fresh weight of the subsamples. These lasts were finally reduced to fine powder for C analysis at the elemental analyser.

### Soil

On the four random selected plots used for the litter’s estimate, one soil core up to 60 cm depth was collected using a petrol driven pneumatic auger (PPA samples; Eijkelkamp, the Netherlands) to quantify organic C content and soil bulk density at different depths (four sampling points per site). Once in the lab, each core was divided into four soil horizons (0–15; 15–30; 30–45; 45–60 cm), air-dried and sieved at 2 mm. Soil bulk density (kg m^− 3^) was calculated for each layer as the ratio between the weight of the sieved soil and the sample total volume. Subsamples of the sieved soil were taken, ball-milled and stored in plastic vials for the further chemical analysis. Before analysis, all soil samples were treated with HCl to eliminate carbonates [47]. Then, C content was measured using a CHNS Elemental Analyser (Vario Microcube, © Elementar).

### Carbon estimation according to standard procedures

The estimation methodologies of each C pool were grouped based on the effort and the amount of required data. In Method 1, no field data were needed but default values were used; in Method 2 and 3, different combinations of tree, deadwood, litter, and soil data were used; in Method 4, only tree DBHs were used as input data (Table 1).

**Table 1:** Summary of methods codes

Compartment	M1	M2	M3	M4
Aboveground biomass	Fixed	BEF x field data	BCEF x field	Allometric equations x field data
Belowground biomass	Fixed	Root: aboveground biomass	Root: aboveground biomass	Allometric equations x field data
Deadwood	Fixed	Field data	Field data	% aboveground biomass
Litter	Fixed	Field data	Field data	GSOC map
Soil	Fixed	Field data	Field data	GSOC map

#### Method 1 (M1)

The default method is based on the application of standard reference values provided by IPCC (2003, 2019) [17, 35] and is usually used in absence of country-specific data, here constitutes a reference point for the following methodologies.

#### Method 2 (M2)

IPCC (2003) [35] allows to estimate living aboveground biomass (AGB) converting merchantable volume (m^3^) to total aboveground biomass (Mg) and then to aboveground C stock (Mg C) using species-specific or type specific (broadleaves/conifers) biomass expansion factors (BEFs). In this study, we summed the leaves/needles C stock to the aboveground C stock (stem and branches). The total C stock for each species *i* (C_i_) in each plot is then equal to:

$${C}_{i}={V}_{i}\times {D}_{i}\times {BEF}_{i}\times {C}_{t\%}+LAI\times {SLM}_{i}\times \frac{{BA}_{i}}{{BA}_{TOT}}\times {C}_{il\%}$$

where V_i_ is the merchantable volume (m^3^), D_i_ is the species-specific basic density (Mg m^− 3^, see par. 2.2.1), BEF_i_ is the species-specific biomass expansion factor, C_t%_ is the type specific C content, SLM_i_ is the specific leaf mass (Mg m^− 2^), LAI is the leaf area index (m^2^), BA_i_ basal area of each species (m^2^) within the plot and BA_TOT_ is the total basal area, C_if%_ is the species-specific leaf carbon content.

Total living C stock was then obtained by adding to the aboveground (AGB) the belowground biomass (BGG) through root to shoot ratios [42].

The other C pools (deadwood, litter, and soil) were obtained from the field data using the procedures described in the previous paragraphs.

#### *Method 3* (M3)

As wood basic density depends on tree growth rate [48], tree age and height, we also compute the standing aboveground biomass by multiplying the wood volume by biomass conversion and expansion factors (BCEFs) as suggested by IPCC (2006) [42]. Again total living C stock was then obtained by adding to the aboveground (AGB) the belowground biomass (BGG) through root to shoot ratios [42]. Deadwood, litter, and soil C stocks were estimated following the same procedure described for Method 2.

#### Method 4 (M4)

Forrester et al. [30] have proposed a large set of global biomass equations for different species and different tree compartments (total aboveground, stem, branches, foliage, roots). In this study, we used the following equation to estimate the biomass of each single tree as it is the only one suitable for all the specie present at our study sites:

$$\text{ln}\left(B\right)=\text{l}\text{n}\left({B}_{0}\right)\times {B}_{1}\times \text{l}\text{n}\left(DBH\right)$$

where B_0_ and B_1_ are species-specific scale factors suggested by the authors and DBH is the diameter at breast height. Total living C stock was obtained by adding to the aboveground (AGB) the belowground C (BGG) obtained applying the root biomass equation suggested by Forrester et al. [30].

Deadwood C stock was estimated applying IPCC (2003) [35] dead to live ratio. More specifically, deadwood is assumed to be 20% and 14% of the living biomass in evergreen and deciduous forests, respectively. Soil and litter were derived by the GSOC map [49], which provides soil C stock up to a depth of 30 cm, including litter.

### Analysis

Analysis of variance (two-way ANOVA) was performed to test for significant differences among all the computing approached. When significant differences were found a post-hoc Bonferroni test was applied. Data were checked for normality of the residuals and homogeneity of variances before performing the analysis. All statistical analyses were performed using R 4.2.2 statistical packages.

## Results

### Aboveground and belowground biomass

None significant statistical difference in standing volume and basal area of living trees as well as in coarse woody debris volume was found between the two study sites (Table 2).

**Table 2:** Living trees volume and basal area and coarse woody debris volume at the two study sites. Mean ± standard error

Site	Living trees volume (m^3^ ha^− 1^)	Living trees basal area (m^2^ ha^− 1^)	Coarse woody debris volume (m^3^ ha^− 1^)
BGO	1022 ± 80	60 ± 4	374 ± 29
PER	994 ± 74	59 ± 4	411 ± 26

**Fig. 2:**
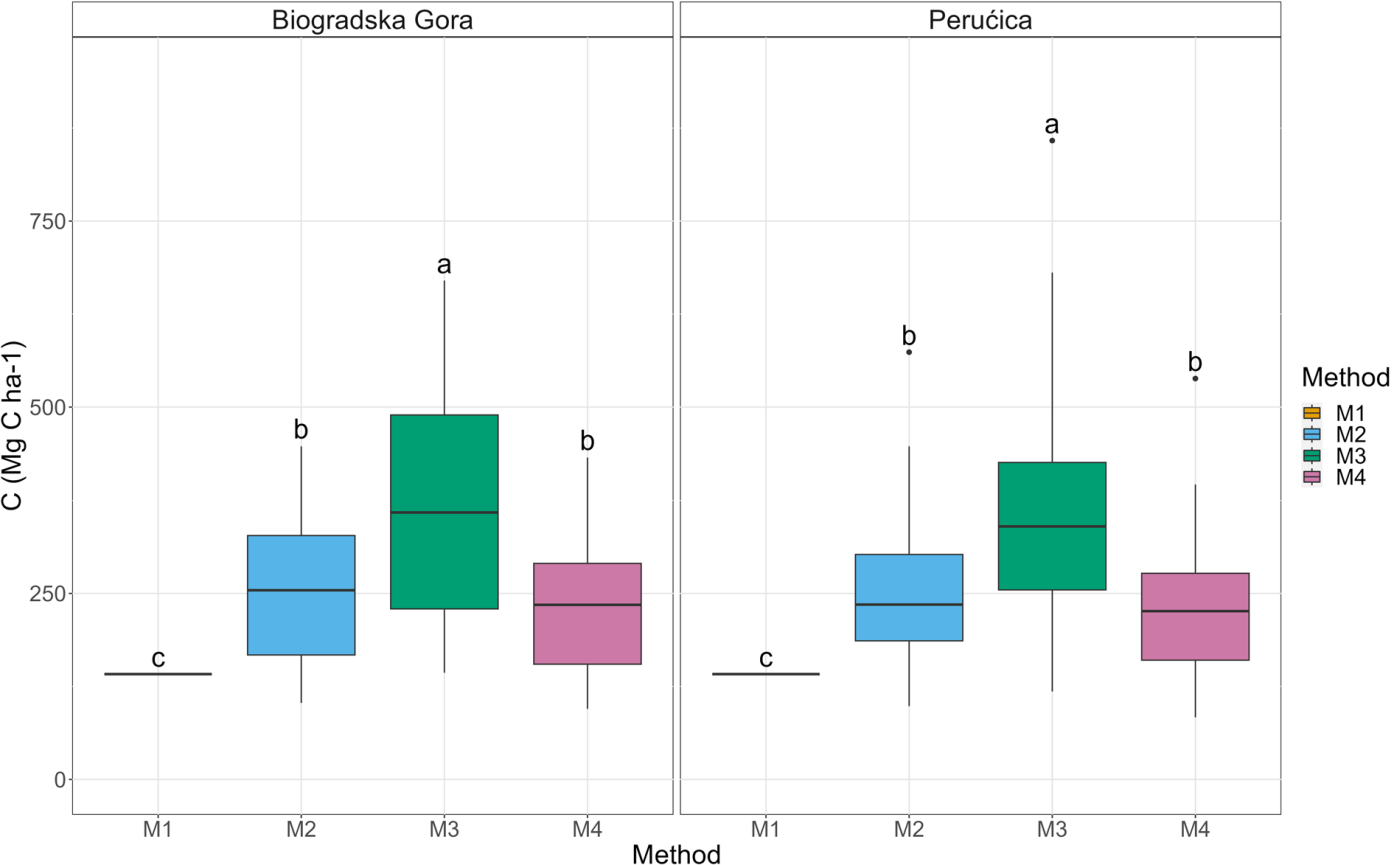
Aboveground C stock computed using the different accounting methodologies. Different letters indicate a significant difference among the adopted methodologies

As well, aboveground biomass C stock (AGBC; method 2) showed no differences between the two sites. On the contrary, significant differences were found among the applied methodologies at both study sites (Fig. 2). Method 3 showed a significantly higher AGBC (367 ± 21 Mg C ha^− 1^) compared to Method 2 (259 ± 14 Mg C ha^− 1^) and Method 4 (235 ± 13 Mg C ha^− 1^). The lowest values were registered using Method 1 for AGBC estimation (142 Mg C ha^− 1^). When different 5-cm diameter classes were considered, a significant difference was only found in the 30 cm class and between Method 4 and all the other methods (data not shown).

**Fig. 3:**
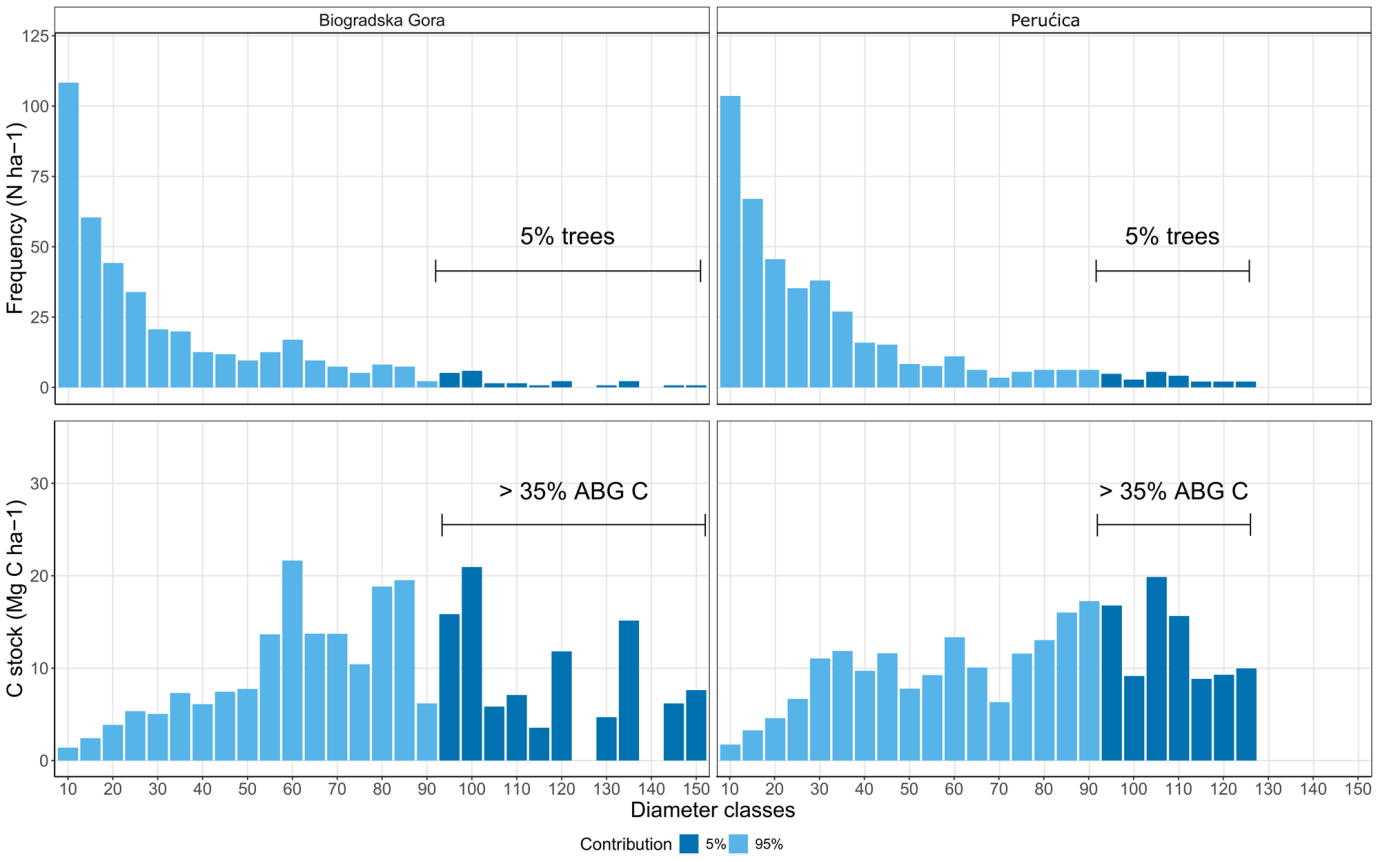
Tree density (N ha-1; above) and C stock M2 (Mg C ha-1; below) distribution in diameter classes

The analysis of the AGBC stock distribution through diameter classes showed that the largest amount of AGBC (more than 35%) was stocked in the 5% of standing trees (DBH > 90 cm, Fig. 3).

**Fig. 4:**
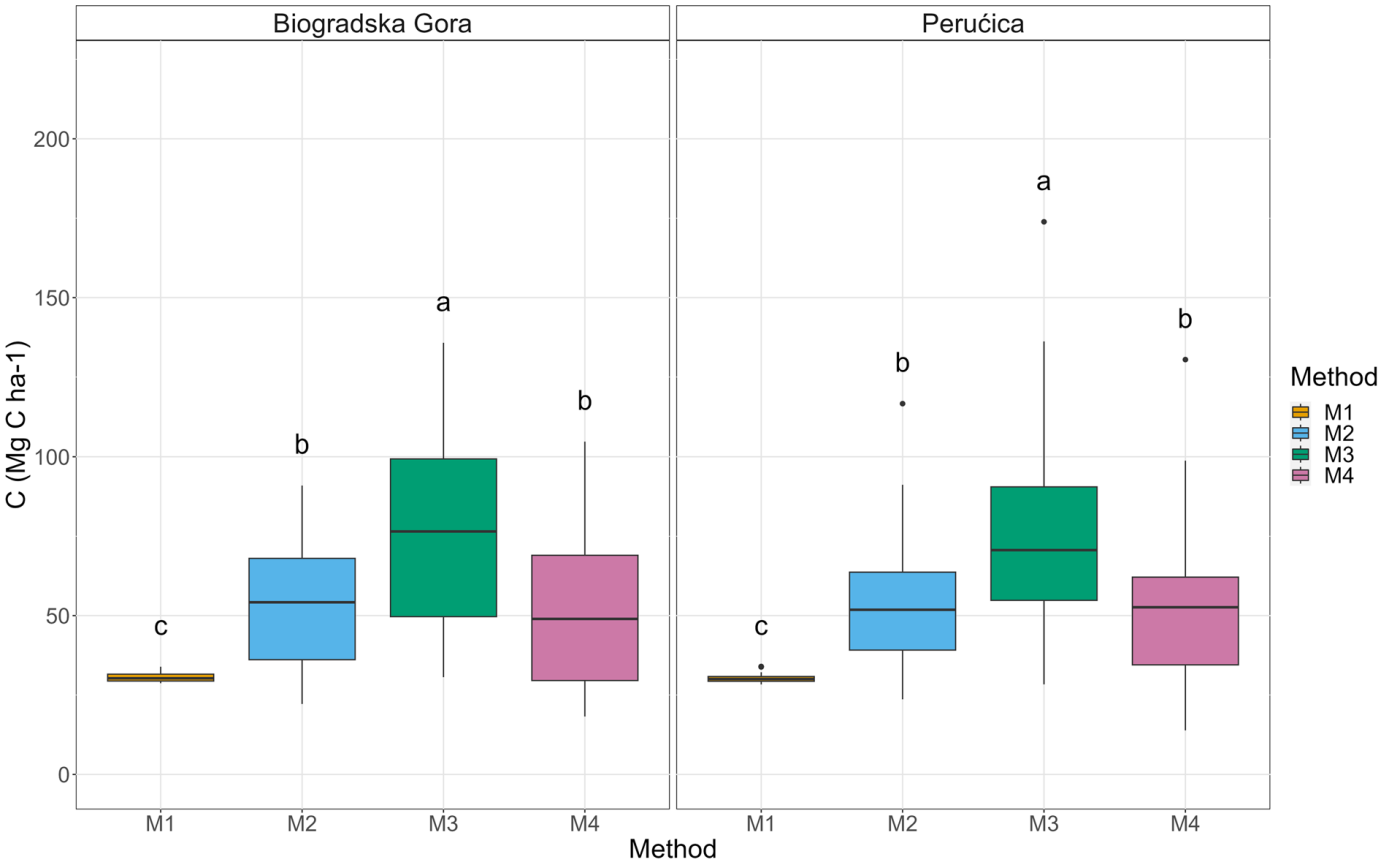
Belowground C stock box-plot. Different letters indicate a significant difference among the adopted methodologies

Because of the variability in AGBC, also belowground biomass C stock (BGBC) presented significant differences among the four methods (Fig. 4) with a higher value in Method 3 (77 ± 4 Mg C ha^− 1^) and lower in Method 2 (55 ± 3 Mg C ha^− 1^), Method 4 (52 ± 3 Mg C ha^− 1^) and Method 1 (31 ± 0 Mg C ha^− 1^).

Total living biomass C stock was obtained adding BGBC to AGBC and, as for the two compartments, significant higher values were shown by Method 3 (444 ± 25 Mg C ha^− 1^) than Method 2 (314 ± 16 Mg C ha^− 1^) and Method 4 (287 ± 16 Mg C ha^− 1^). The lowest was again Method 1 (172 ± 2 Mg C ha^− 1^).

### Dead biomass: deadwood and litter

Laboratory analysis on deadwood samples provided the C content (%) and basic wood density (g m^− 3^) for each decay stage. In BGO, similar C content was obtained for the first four classes (min 46.07% – max 47.97%) and higher values for the most decomposed one (54.98%). On the contrary, in PER lower content was found in the first class (45.94%) and then growing in the following decay classes (48.00-50.63%-51.62%) except for the last one (48.19%). An opposite trend was shown by basic wood density. In fact, at both sites, it decreased from the first to the last class (BGO from 0.53 to 0.27 g cm^− 3^, PER from 0.45 to 0.12 g cm^− 3^), only BGO class 5 had a slightly higher value than the previous one. Applying these values to estimated deadwood volume (M2 and M3), an average C stock of 54.0 ± 3.4 Mg C ha^− 1^ was obtained (17% of living biomass C). At both sites, lying deadwood (logs) represented around 2/3 of the total deadwood biomass and snags 1/3, stumps represented a marginal C pool in both old-growth forests (1.4% in BGO and 1.5% in PER).

**Fig. 5:**
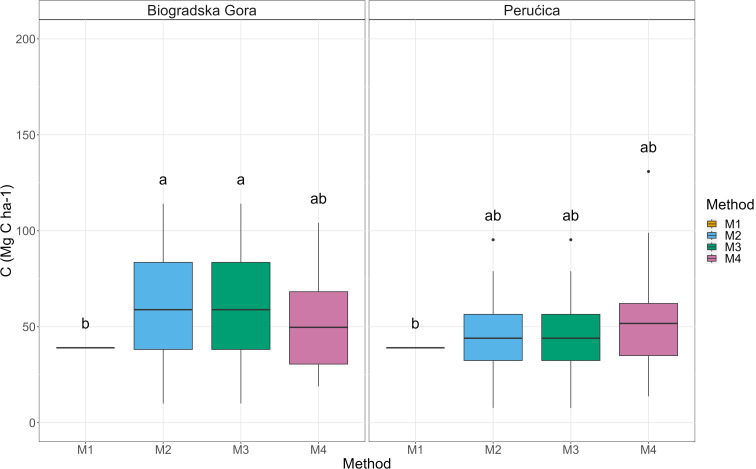
Deadwood C stock. Different letters indicate a significant difference among the adopted methodologies

Regarding the distribution of the C stock in the decay classes, in BGO the third and the fourth classes reached almost 70% (40.6% and 28.1%, respectively) of the total C while 7.1% of the C was in class 1, 14.4% in class 2 and 9.7% in class 5. Similarly, in PER class 3 and 4 represented the main accumulation stages with almost the 80% (49.0% and 30.6%) of the total, while 2.4% of the C was in class 1, 11.6% in class 2 and 6.4% in the most decomposed one. Regarding the deadwood C stock significant differences were found both among methods and between the two sites (Fig. 5). More specifically, significant lower values were reported by Method 1 (40 Mg C ha^− 1^) in comparison to the others (M2-3: 54 ± 3 Mg C ha^− 1^; M4: 52 ± 3 Mg C ha^− 1^).

As for dead wood, litter C stock measured in the field (M2 and M3) were significantly different from IPCC derived estimates (M2-3: 5 ± 0.5 Mg C ha^− 1^ vs. M1: 21 Mg C ha^− 1^, respectively).

### Soil

**Fig. 6:**
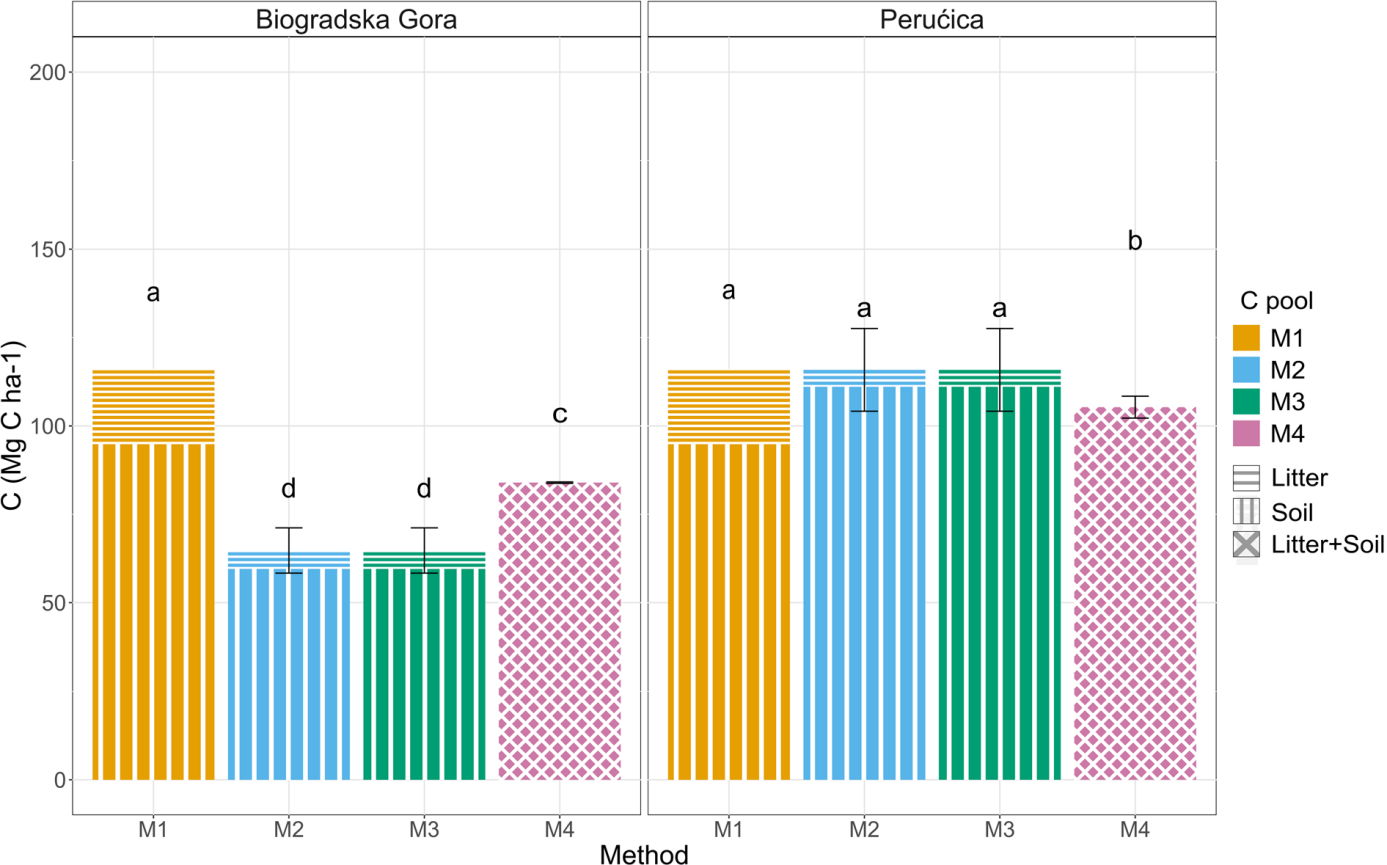
Litter and soil (0–30 cm) C stock. Different letters indicate a significant difference among the adopted methodologies

To compare field data and standard value (i.e., IPCC reference values or Global Soil Carbon map), only the upper 30 cm of soil were considered. Soil C stock measured in the field (M2 and M3: 86 ± 11 Mg C ha^− 1^) was significantly different than the IPCC default value (M1: 95 Mg C ha^− 1^). Significant differences were also found between the two forests with higher values in Perućica (PER M2-3: 111 ± 15 Mg C ha^− 1^, BGO M2-3: 60 ± 10 Mg C ha^− 1^). By adding litter C stock to soil data, it was possible to compare the field data with the Global Soil Organic Carbon map (M4) and no significant differences were detected between them (M2-3: 91 ± 3 Mg C ha^− 1^ vs. M4: 95 ± 2 Mg C ha^− 1^, respectively). As for soil C stock, significant differences were found between the two study areas (PER M4: 105 ± 3 Mg C ha^− 1^, BGO M2-3: 84 ± 0.2 Mg C ha^− 1^; (Fig. 6).

Anyway, soil samples were entirely analysed, down to 60 cm, and an additional C stock of 35 Mg C ha^− 1^ and 62 Mg C ha^− 1^, in BGO and PER respectively, need to be considered.

### Total C stock

Summarising and comparing previous paragraphs data by method, significant difference can be noted (Table 3). Indeed, Method 1 reported the minimum values (328 Mg C ha^− 1^) significantly lower than Method 2 (average 459 ± 13 Mg C ha^− 1^) and Method 4 (average 435 ± 5 Mg C ha^− 1^). Maximum and significant higher value was shows by Method 3 (average 589 ± 11 Mg C ha^− 1^).

**Table 3:** C stock estimation summary

C pool	M1		M2		M3		M4	
	**BGO**	**PER**	**BGO**	**PER**	**BGO**	**PER**	**BGO**	**PER**
Aboveground biomass	142	142	263 ± 20	255 ± 18	372 ± 31	362 ± 28	240 ± 19	231 ± 18
Belowground biomass	31	31	56 ± 4	54 ± 4	79 ± 6	76 ± 6	53 ± 5	52 ± 5
Deadwood	40	40	62 ± 6	46 ± 3	62 ± 6	46 ± 3	53 ± 5	52 ± 5
Litter	21	21	5 ± 1	5 ± 0.3	5 ± 1	5 ± 0.3	84 ± 0.2	105 ± 3
Soil (30 cm)	95	95	60 ± 10	111 ± 15	60 ± 10	111 ± 15		
Tot	328	328	446 ± 41	471 ± 40	578 ± 54	600 ± 55	430 ± 29	440 ± 31

## Discussion

### Carbon stock in forests

The estimation of C pools using different methodologies confirms our initial hypothesis that temperate old-growth forests contain one of the largest C stock (C stock ha^− 1^) in Europe and those forests located in the Dinaric Alps are the most prominent [50]. BGO had a total C stock of 481 ± 51 Mg C ha^− 1^ (66% in living biomass, 14% in dead biomass and 20% in soil down to 60 cm; M2); PER reached 533 ± 46 Mg C ha^− 1^ (59% in living plants, 9% in dead biomass and 32% in soil down to 60 cm; M2). These values are indeed higher than what has been reported so far in other European old-growth temperate forests (Table 4). Seedre et al. [23] estimated the C stock in a montane Norway spruce (*Picea abies* Karst.) old-growth forest in the Bohemian Forest (Czech Republic) reporting 393 ± 92 Mg C ha^− 1^ of total C stock, out of which 207 ± 59 Mg C ha^− 1^ (53%) were in living biomass pool, 15 ± 9 Mg C ha^− 1^ (4%) in the dead biomass and 171 ± 49 Mg C ha^− 1^ (43%) into the soil. In Seedre et al. [23], dead roots and soil down to bedrock were also included. In Bialowieza core area, Matuszkiewicz et al. [21] reported an overall C stock equal to 323 Mg C ha^− 1^ divided in 117 Mg C ha^− 1^ (36%) in living biomass, 20 Mg C ha^− 1^ (6%) in deadwood and 186 Mg C ha^− 1^ (58%) in soil (up to 1 m depth). When compared to IPCC estimates for European mountain temperate primary forest (Method 1) [17], the total C stock (328 Mg C ha^− 1^) is still lower than our estimates. Keeton et al. [20] study on Carpathian Norway spruce-silver fir old-growth forest analyses only the aboveground C pool and reports a variable range of 155–165 Mg C ha^− 1^ that is much lower than the results of our study (M2: BGO 263 Mg C ha^− 1^ and PER 255 Mg C ha^− 1^). Finally, lower data were also reported by Petritan et al. [22] in their research on the deadwood compartment in a beech-silver fir mixed virgin European forest in the Southern Carpathian. Indeed, their show an average amount of 16 Mg C ha^− 1^.

**Table 4:** Total C stocks at the two study sites according to the different applied accounting methods and reference values from previous studies

C pool	M1		M2		Seedre et al. [23]	Matuszkiewicz et al. [21]	Ķēniņa et al. [51]
	**BGO**	**PER**	**BGO**	**PER**			
Living biomass	173	142	319 ± 24	309 ± 22	207 ± 59	117	171 ± 6
Dead biomass	60	39	67 ± 7	51 ± 3	15 ± 9	20	15 ± 2
Soil	95 (30 cm)	95 (30 cm)	95 ± 20 (60 cm)	173 ± 21 (60 cm)	171 ± 49 (down to bedrock)	186 (100 cm)	105 ± 46 (80 cm)
Tot	328	328	481 ± 51	533 ± 46	393 ± 92	323	291 ± 54

The comparison among our study and previous ones in other European biomes confirm the highest C stock reached by temperate forests. Ķēniņa et al. [51] in hemiboreal Scots pine Latvian old-growth forests reported 171 ± 6 Mg C ha^− 1^ (59%) in living biomass, 15 ± 2 Mg C ha^− 1^ (5%) in deadwood and 105 ± 46 Mg C ha^− 1^ (36%) in soil down to 80 cm (total C stock: 291 ± 54 Mg C ha^− 1^).

Moreover, our study confirm the different distribution of C stocks into the different ecosystem pools in the old-growth forests [52]. In fact, while at global scale soil has been reported to be the most important C pool in forest ecosystems [53, 54], living biomass is storing the largest amount of C stock in old-growth forests (53–66%, on average).

Managed European forests contain three-times less C than old-growth forests (170 Mg C ha^− 1^; [3]) and store more C into soil than in plants (54% vs. 36%). Thus, old-growth forests represent a very important reference point for managed forests to optimize their potential C sink. In this context, our work sets a benchmark against which evaluating the C sequestration potential of temperate managed forests [55, 56].

Our research also suggests that the larger amount of C stocked in living biomass in old-growth forests is related to the presence of few larger trees (35–37% of C is stored in 5% of living trees; Fig. 3). Other authors have previously reported similar observations [57–62]. For example, in Lutz et al. [57] 50% of living biomass C is stocked in 1% of trees; in Mildrexler et al. [59] 3% of trees contain 42% of living biomass C; in Slik et al. [61] the 2–4% of trees contain the 25–45% of biomass. Apart for C sequestration, large-diameter trees play also a crucial role in biodiversity conservation, they host a diversity of tree-related microhabitats [63] and species missing in small-diameter trees of managed forests [64, 65].

### Carbon stock estimation methodologies

The research’s second aim was to compare different methods to estimate C stocks to verify their suitability for old-growth forests and the potential different applicability depending on the considered compartment. For aboveground and belowground C stock, Method 4 (i.e., using equations developed in managed forests) showed an excellent agreement with the estimates obtained using Method 2 (our reference). On the contrary, Method 1 and 3 showed significant lower and higher estimates, respectively. When moving to the deadwood compartment, field estimates (Method 2 and 3) showed higher values than Method 1 or Method 4. Anyway, live to dead ratio of Methods 2–3 and Method 4 was comparable (about 18%) and, also the underestimation, was no significant. Contrary, Method 1 deeply underestimated deadwood C stock. In addition to the methods here applied, IPCC guidelines proposes a third approaches based on field measurement for deadwood volume estimation and the using of a fixed conversion factor (0.5 Mg C (Mg d.m.)^−1^). This, in our study lead to minimum over/underestimation in BGO and in PER.

Similarly, there was a significant difference between litter field data (Method 2 and 3) and IPCC standard value (Method 1 and 4). As for deadwood, IPCC proposed an alternative method suggesting the use of 0.37 g C/g as C factor to convert litter biomass (Mg) in C. Our laboratory analysis on litter samples provides an average C content of 0.44 g C/g, therefore the use of IPCC carbon factor has leaded to an average underestimation of 16%.

Remarkable differences were also noted in soil C stock both between the two study sites and among the estimation methods. These differences are probably related to the fact that local conditions strongly affect soil C stocks and IPCC default values are not suitable to properly represent this compartment. On the contrary, GSOC map reports very similar values to those derived from our field survey in the two old-growth forests.

## Conclusions

The current study represents a starting point for future research and analysis about carbon storage and fluxes of the old-growth forests in the Balkans. We demonstrated that, especially for ecosystem type, field data would give most precise insight of C stock (and eventually fluxes) than general estimation methods. According to our results, living biomass is the more relevant C pool in these ecosystems, but it is also the one with the major potential estimation errors due to the use of many general parameters such as basic wood density, biomass expansion factor, and C content, all parameters that are influenced by climatic and environmental conditions. More specifically, biomass expansion factors are the ones presenting the major variability as they are size and age-dependent and are normally developed using small and medium diameter plants. However, in managed forests BEFs have been shown to be able to estimate forest biomass with a good approximation at European scale [54]. Moreover, many approaches exist for aboveground compartment and the most suitable can be selected time by time. Conversely, other C pools such as litter and soil have no alternative to field data collection. Global Soil Organic Carbon (GSOC) map, as here demonstrate, provides more reliable results than IPCC default values but it is limited to the topsoil (30 cm) which is too shallow when considering old-growth forest soil [53, 54]. Evidence of this was also found in current study, indeed in BGO and PER the topsoil represents only the 63% and 64% respectively of total soil C stock explored (60 cm), so future studies are needed to improve to extend the knowledge about soil C pool. Finally, for deadwood C stock, where such field data are not available, IPCC dead: live ratio represents a very predictable option.

Old-growth forests may store large amount of C for centuries, but they are vulnerable ecosystems to the on-going climate change due to their greater sensibility to drought and natural disturbances which will be more frequent in the future [66–69]. Moreover, their overall contribution to climate mitigation are limited by the fact that they represent only 1% of temperate forests [5] and despite the prominent C stock, C sink is low [70, 71]. Nevertheless, old-growth forests constitute a crucial reference point for close-to-nature silviculture in managed forests, and a benchmark for forest carbon stock. The development of further and deeper research about the carbon stock and fluxes in old-growth stands is of primary importance to understand potentiality and limits of the climate mitigation role of the forests.

## Data Availability

The data that support the findings of this study are available from the corresponding author upon reasonable request.

## Abbreviations

AGB: Aboveground biomass
AGBC: Aboveground biomass C stock
BEF: Biomass expansion factor
BEFC: Biomass conversion and expansion factor
BGB: Belowground biomass
BGBC: Belowground biomass C stock
BGO: Biogradska Gora
C: Carbon
DBH: Diameter at breast height
GHG: Greenhouse gases
IPCC: Intergovernmental Panel on Climate Change
LAI: Leaf area index
M1: Method 1
M2: Method 2
M3: Method 3
M4: Method 4
PER: Perućica
