# Correction to: The largest European forest carbon stocks are in the Dinaric Alps old-growth forests: comparison of direct measurements and standardised approaches

**DOI:** 10.1186/s13021-024-00266-0

**Published:** 2024-06-27

**Authors:** Alessia Bono, Giorgio Alberti, Roberta Berretti, Milic Curovic, Vojislav Dukic, Renzo Motta

**Affiliations:** 1 Department of Agricultural, Forest and Food Sciences (DISAFA), University of Turin, Largo Paolo Braccini 2 - IT, Grugliasco, TO 10095 Italy; 2 Department of Agricultural, Food, Animal and Environmental Sciences, University of Udine, Via delle Scienze 206 - IT, Udine, UD 33100 Italy; 3 Biotechnical Faculty, University of Montenegro, Mihaila Lalica 1, Podgorica, Montenegro; 4 University of Banja, Luka, blv. Stepa Stepanović, 75, Banja Luka, 78000 Republic of Srpska

**Correction to: Carbon Balance and Management (2024) 19:15**

10.1186/s13021-024-00262-4

Following publication of the original article [1], the authors identified errors in the article title and in the author group. These errors have been updated with this correction.

The article title “The largest European forest carbon stocks are in the Dinaric Alps old-growth forests: comparison of direct measurements and standardised approaches” was incorrectly written as “The largest European forest carbon sinks are in the Dinaric Alps old-growth forests: comparison of direct measurements and standardised approaches”.

The given and family names of the authors “Alessia Bono, Giorgio Alberti, Roberta Berretti^1^, Milic Curovic, Vojislav Dukic and Renzo Motta” were incorrectly structured as “Bono Alessia, Alberti Giorgio, Berretti Roberta, Curovic Milic, Dukic Vojislav and Motta Renzo”.

The original article has been corrected.

## References

[1] Bono A, Alberti G, Berretti R (2024). The largest European forest carbon stocks are in the Dinaric Alps old-growth forests: comparison of direct measurements and standardised approaches. Carbon Balance Manage.

